# Cleavage of the pseudoprotease iRhom2 by the signal peptidase complex reveals an ER-to-nucleus signaling pathway

**DOI:** 10.1016/j.molcel.2023.12.012

**Published:** 2024-01-05

**Authors:** Iqbal Dulloo, Michael Tellier, Clémence Levet, Anissa Chikh, Boyan Zhang, Diana C. Blaydon, Catherine M. Webb, David P. Kelsell, Matthew Freeman

**Affiliations:** 1 Dunn School of Pathology, University of Oxford, South Parks Road, Oxford OX1 3RE, UK; 2 Blizard Institute, Faculty of Medicine and Dentistry, Queen Mary University of London, Newark Street, London E1 2AT, UK

## Abstract

iRhoms are pseudoprotease members of the rhomboid-like superfamily and are cardinal regulators of inflammatory and growth factor signaling; they function primarily by recognizing transmembrane domains of their clients. Here, we report a mechanistically distinct nuclear function of iRhoms, showing that both human and mouse iRhom2 are non-canonical substrates of signal peptidase complex (SPC), the protease that removes signal peptides from secreted proteins. Cleavage of iRhom2 generates an N-terminal fragment that enters the nucleus and modifies the transcriptome, in part by binding C-terminal binding proteins (CtBPs). The biological significance of nuclear iRhom2 is indicated by elevated levels in skin biopsies of patients with psoriasis, tylosis with oesophageal cancer (TOC), and non-epidermolytic palmoplantar keratoderma (NEPPK); increased iRhom2 cleavage in a keratinocyte model of psoriasis; and nuclear iRhom2 promoting proliferation of keratinocytes. Overall, this work identifies an unexpected SPC-dependent ER-to-nucleus signaling pathway and demonstrates that iRhoms can mediate nuclear signaling.

## Graphical abstract

**Graphical abstract:**
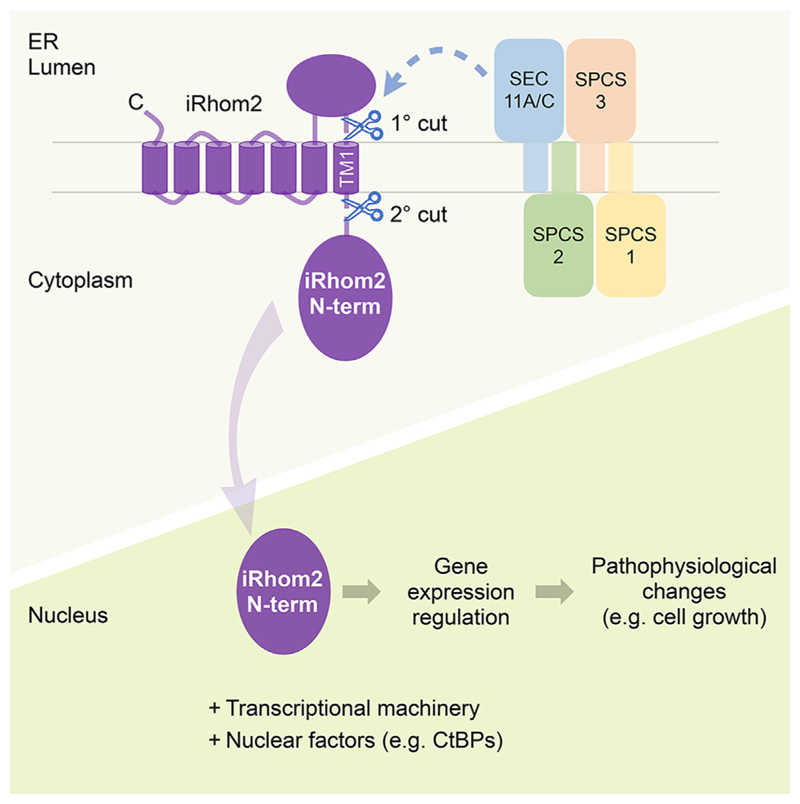

## Introduction

The rhomboid-like superfamily of membrane proteins comprises the originally discovered rhomboid intramembrane serine proteases and multiple pseudoproteases that, despite being widely conserved, have lost their protease activity.^1,2^ Pseudoenzymes were once assumed to be functionally dead evolutionary remnants, but they are emerging as an important class of proteins with significant biological functions,^3,4^ and this is consistent with what is known of the diverse functions of pseudoprotease members of the rhomboid-like superfamily, the best characterized of which are iRhom1 and iRhom2.^5^ They are now most famous as regulatory cofactors of ADAM17, a cell surface metalloprotease responsible for the release of important intercellular signaling proteins.^6^ As such, iRhoms control both inflammatory signaling by the cytokine TNF and growth factor signaling by members of the EGF family.^7–9^ iRhoms are, however, multifunctional, and they also participate in, for example, the response to chronic endoplasmic reticulum (ER) stress^10^ and viral infection.^11^

Rhomboid-like proteins have a modular structure.^5^ The mechanistic theme believed to underlie all their functions is the specific recognition of, and interaction with, transmembrane domains (TMDs) of substrates and client proteins. This is mediated by their conserved transmembrane core. All rhomboid-like proteins also have cytoplasmic and luminal/extracellular domains, which are less well conserved, and are presumed to mediate functions more specific to particular members of the superfamily. In the case of the iRhoms, these include a long cytoplasmic N terminus, and a large luminal loop between TMD1 and TMD2, called the iRhom homology domain (IRHD).^12^ The cytoplasmic N terminus is predicted to be largely unstructured but has important regulatory properties, interacts with several accessory factors and signaling proteins, and is the site of post-translational modifications^13–15^ and disease-associated mutations.^16^

We and others have previously noted that C-terminally tagged iRhoms exist in two forms: the full-length protein and a shorter fragment^7–9,13,14,17,18^ whose size suggests that the N-terminal cytoplasmic domain is deleted. Indeed, the N-terminal domain may not be essential for some iRhom functions: its deletion is reported not to abolish function but instead to cause elevated constitutive ADAM17 activity in mammalian cells,^19,20^ and in *Drosophila*, a wing phenotype that suggests hyperactivity.^18^ Nevertheless, whether shorter forms of iRhoms have any physiological function remains unexplored.

Pursuing this question, we have discovered that endogenous iRhom2 undergoes partial proteolytic cleavage to generate three stable forms—the full-length protein and both N- and C-terminal fragments. We have identified the protease responsible for this iRhom2 cleavage as the signal peptidase complex (SPC). SPC is primarily the protease responsible for the removal of signal peptides from proteins entering the ER,^21,22^ but in this case, it cleaves iRhom2 non-canonically, adjacent to its first TMD. The consequence of the primary cleavage by SPC coupled with secondary proteolytic processing is the release of the N-terminal domain of iRhom2 and its translocation to the nucleus, where it modifies the cellular transcriptome. Nuclear iRhom2 associates with transcriptional repressors C-terminal binding proteins 1 and 2 (CtBP1/2) to downregulate the expression of some genes. Skin biopsies from patients with psoriasis or the genetic syndrome tylosis with oesophageal cancer (TOC) or non-epidermolytic palmoplantar keratoderma (NEPPK) all show elevated nuclear iRhom2. Among other targets, nuclear iRhom2 regulates the gene expression of cytoskeletal scaffolding protein K16 and promotes cellular proliferation of keratinocyte cells. Overall, this work demonstrates the ability of SPC to modulate signaling by cleaving iRhoms as non-canonical substrates.

## Results

### A cleaved N-terminal fragment of iRhom2 translocates to the nucleus

**Figure 1:**
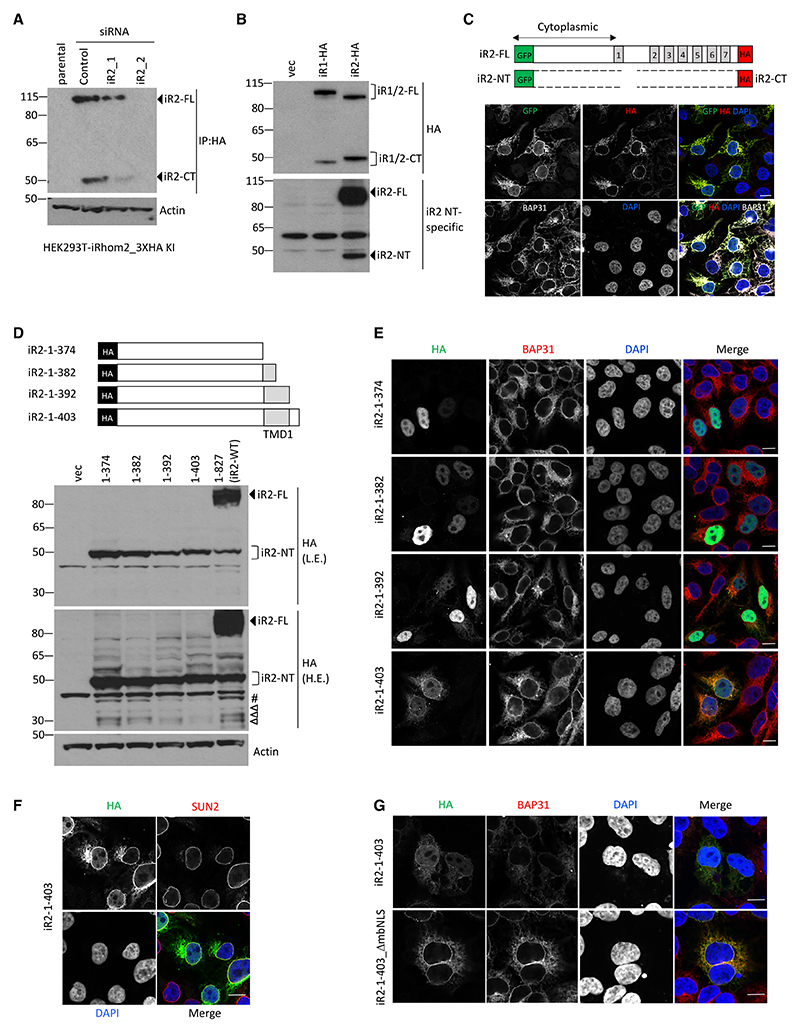
Cleaved iRhom2 is nuclear (A) Endogenous iRhom2 proteins were detected in HEK293T cells with knockin of 3XHA tag at C-terminal of iRhom2 gene locus. Due to low expression, iRhom2 was first immunoprecipitated (IP: HA), followed by immunoblotting with HA antibody. Cells were transfected with control siRNA or two independent iRhom2 siRNAs to confirm specificity. (B) iRhom1 and iRhom2 proteins were analyzed by immunoblotting in HEK293T cells transfected with indicated constructs using either HA or iRhom2 N-terminal specific antibody. (C) Schematic showing expected products from GFP-iRhom2-3XHA (top). Immunofluorescence of GFP-iRhom2-3XHA transfected in HEK293T cells. Cells were stained for GFP (green), HA (red), BAP31 (white) as ER marker, and DAPI (blue). Scale bars, 10 μm. (D) Schematic showing truncated iRhom2 mutants with N-terminal 2XHA tag (top). iRhom2 proteins were analyzed in HEK293T cells transfected with indicated constructs by immunoblotting using HA antibody (bottom). L.E, low exposure; H.E, high exposure. Empty triangles denote further proteolytically processed soluble nuclear iRhom2 proteins. # denotes largest soluble nuclear iRhom2 detected. (E) Immunofluorescence of truncated iRhom2 mutants transfected in HEK293T cells. Cells were stained for HA (green), BAP31 (red), and DAPI (blue). Scale bars, 10 μm. (F and G) Immunofluorescence of iR2-1-403 (F and G) and iR2-1-403_ΔmbNLS (G) transfected in HEK293T cells. Cells were stained for HA (green), either SUN2 as nuclear membrane marker (red) (F) or BAP31 (red) (G) and DAPI (blue). Scale bars, 10 μm.

A HEK293T cell line in which endogenous iRhom2 was C-terminally tagged with a 3XHA tag by CRISPR knockin confirmed previous observations^13^ that endogenous human iRhom2 exists as both full-length and shorter C-terminal forms. The identity of both bands as iRhom2 was validated with two different siRNAs against iRhom2 (Figure 1A). Similarly, overexpression of C-terminally 3XHA-tagged human iRhom1 and mouse iRhom2 led to the generation of both full-length protein (±100 KDa; iR1/2-FL) and a shorter C-terminal form of approximately 50 kDa (iR1/2-CT) (Figure 1B, top). Using an antibody specific to the mouse N-terminal cytoplasmic domain (Figure 1B, bottom), we detected an N-terminal form of iRhom2 of approximately 45 kDa (iR2-NT). The endogenous existence of this N-terminal fragment was confirmed in wild-type (WT) but not iRhom2 knockout mouse lung tissue (Figure S1A). We conclude that both human and mouse iRhoms exist in three major forms—the full-length protein and N- and C-terminal fragments, whose combined sizes suggest that they are products of proteolytic cleavage.

Treatment of cells overexpressing mouse iRhom2 (the default iRhom2 protein used in this work, unless otherwise indicated) with proteasomal inhibitor MG-132 (Figure S1B) or lysosomal inhibitors chloroquine and 3-MA (Figure S1C) did not prevent the generation of the shorter C-terminal iRhom2 fragment, suggesting that the fragments were not protein degradation products. Cycloheximide chase experiments showed that the cleaved iRhom2 protein had a half-life >4 h, whereas the full-length protein half-life was approximately 2 h (Figures S1D and S1E). These data confirm that iRhoms proteins exist in multiple stable forms, apparently due to the proteolytic cleavage of the full-length protein into an N-terminal and a C-terminal fragment.

Full-length iRhom2 is a polytopic membrane protein with two known major cellular locations—the ER and the plasma membrane.^5,13,15^ N-linked glycans attached to ER proteins are sensitive to the deglycosidase endoglycosidase H (Endo H), whereas the glycans on post-Golgi proteins are insensitive to Endo H but can be removed by peptide-*N*-glycosidase F (PNGase F). In cells expressing N-terminally tagged iRhom2, Endo H treatment caused a partial downshift of the full-length iRhom2 fragment, corresponding to the two known locations of full-length iRhom2: in the ER and post-Golgi.^7^ In contrast, the cleaved N-terminal iRhom2 fragment was unaffected by either treatment (Figure S1F), indicating that it was not glycosylated. Integrin Alpha V membrane protein served as a control for the deglycosylation assay. Consistent with the expected locations, immunofluorescent staining showed full-length (GFP and HA tagged) and C-terminal iRhom2 fragment (HA tagged) to be co-localized with the ER-resident protein BAP31, clearly visible in the ER and the nuclear envelope (which is contiguous with the ER) (Figure 1C). To our surprise, however, the N-terminal fragment (iR2-NT), labeled with the GFP tag, also showed low-level but reproducible diffuse staining in the nucleus (Figure 1C). A different iRhom2 construct, tagged with HA at the N-terminal, showed similar nuclear localization (Figure S1G). These data raised the possibility that after cleavage, iR2-NT translocates to the nucleus.

To explore the characteristics of iRhom2 cleavage, we assessed the size of iR2-NT, by expressing different deletion mutants of 2XHA tagged N-terminal domain. The N-terminal iRhom2 cleavage fragment was sized closest to iR2-1-403, which included predicted TMD1 and a few luminal amino acids (Figure 1D). A similar observation was made for human iRhom1 with deletion mutant iR1-1-433 (Figure S1H), indicating that the cleavage occurs around the luminal border of TMD1, which would generate a fragment that remains membrane tethered. Several smaller-sized fragments were also observed (Figure 1D), indicating likely further proteolytic cleavage of iR2-1-403. The intrinsic nuclear localization of the N-terminal domain was confirmed with immunofluorescent staining: iR2-1-374 and iR2-1-382 (Figure 1E) and iR1-1-404 (Figure S1J), all of which lack TMD1, were solely nuclear. In contrast, iR2-1-392, iR2-1-403 (Figure 1E), and iR1-1-433 (Figure S1J), all containing TMD1, showed both soluble nuclear and ER staining, including in the nuclear envelope, with iR2-1-403 (Figure 1F) and the naturally cleaved iR2-NT fragment (Figure S1I) colocalizing with the nuclear envelope marker SUN2. Consistent with these observations, we identified two conserved potential nuclear localization signal (NLS) motifs in the iRhom2 N terminus—one monopartite (mNLS) and one bipartite (bNLS) (Figure S1K). The trafficking of membrane proteins with bulky cytoplasmic domains to the nuclear envelope is dependent on the presence of disordered regions and an NLS^23,24^; in accordance with this, deletion of both NLS motifs from iR2-1-403 markedly reduced soluble nuclear staining (Figure 1G).

Overall, these results led us to conclude that proteolytic release of the N-terminal domain from the full-length ER-localized iRhom proteins leads to their nuclear translocation. The N-terminal fragments contain TMD1 and thereby remain membrane tethered. However, the diffuse nucleoplasmic staining of these fragments from full-length iRhom proteins combined with the solely diffuse nucleoplasmic staining of constructs without TMD1 implies the existence of further proteolytic events that release soluble nuclear iRhom protein fragments into the nucleoplasm.

### iRhom2 undergoes primary cleavage in the luminal juxtamembrane region of TMD1

**Figure 2:**
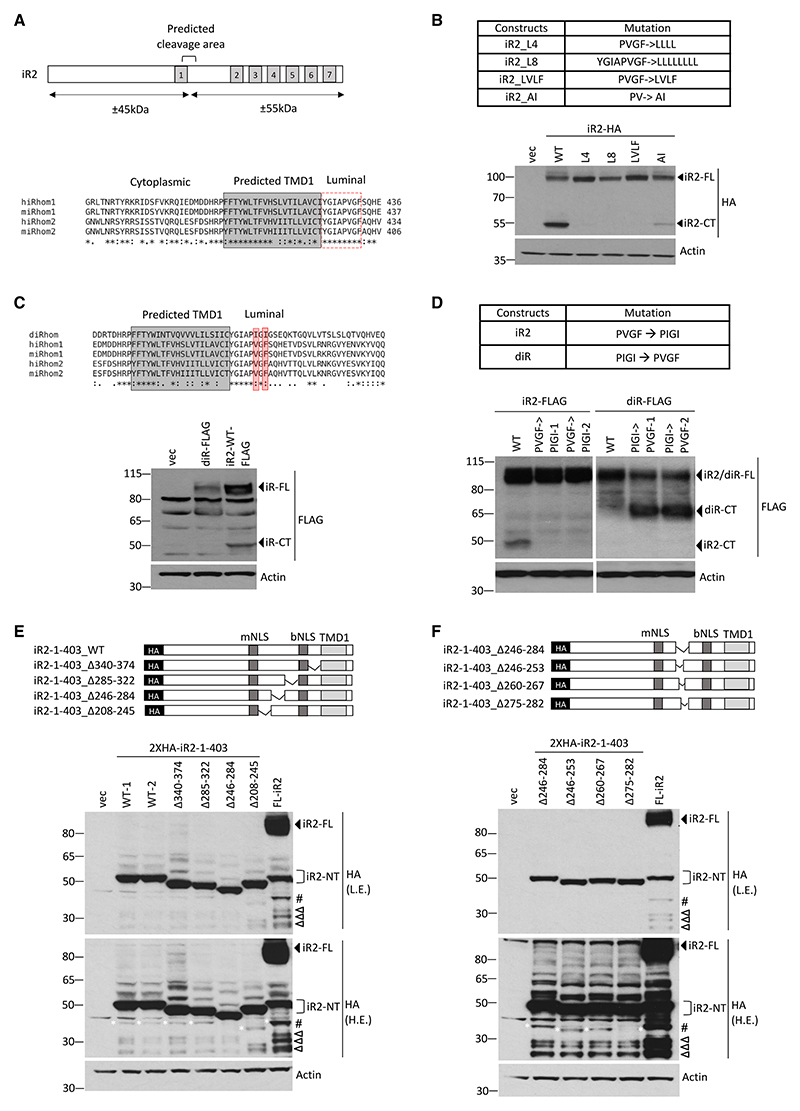
iRhom2 is primarily cleaved within the luminal domain (A) Schematic showing predicted cleavage area of iRhom2 (top). Alignment of protein sequences of iRhom1 and iRhom2 from *Homo sapiens* (h) and *Mus musculus* (m). Boxed sequences are predicted TMD1 (gray) and highly conserved region juxtamembrane of predicted TMD1 (dashed red). (B) Wild-type and various mutated iRhom2 proteins (top) within conserved predicted cleavage region were analyzed in HEK293T cells transfected with indicated C-terminally tagged constructs (bottom) by immunoblotting with HA antibody. (C) Alignment of iRhom proteins sequences from *Homo sapiens* (h) and *Mus musculus* (m) and *Drosophila melanogaster* (d). Boxed sequences are predicted TMD1 (gray) and two amino acids different in *Drosophila* within this highly conserved region of mammalian iRhoms (shaded red) (top). iRhom proteins were analyzed in HEK293T cells transfected with C-terminally tagged *Drosophila* iRhom (diR-FLAG) and iRhom2 (iR2-WT-FLAG) by immunoblotting with FLAG antibody (bottom). (D) iRhom2 and *Drosophila* iRhom mutated within PVGF and PIGI region (top) were analyzed in HEK293T cells transfected with indicated constructs by immunoblotting with FLAG antibody (bottom). 1 and 2 denote two different constructs for same mutations. (E and F) Schematics showing internal deletion mutants of 2XHA-iR2-1-403 (top) and their expression in HEK293T cells transfected with indicated constructs detected by immunoblotting with HA antibody (bottom). L.E, low exposure; H.E: high exposure. Empty triangles denote further proteolytically processed soluble nuclear iRhom2 proteins. # denotes largest soluble nuclear iRhom2 detected.

The initial cleavage of iRhom2 is predicted to be near the luminal end of TMD1 (Figures 1D and 2A), the boundaries of which were further defined based on secondary structure and TMD prediction analyses (Figures S2A and S2B). Sequence alignment of iRhom1 and iRhom2 from mice and humans showed a highly conserved stretch of 8 amino acid residues (YGIAPVGF) in this region (Figure 2A). Mutation to leucine of all (L8), or only the last 4 (L4), of these 8 amino acids abolished cleavage of iRhom2 (Figure 2B), as did the mutation of PVGF to LVLF. In contrast, mutation of PV to AI only partially inhibited cleavage (Figure 2B). We conclude that the PVGF motif is required for iRhom2 cleavage. Significantly, *Drosophila* iRhom does not contain the PVGF motif (PVGF being replaced by PIGI; Figure 2C), and we detected no cleavage of *Drosophila* iRhom (Figure 2C). Furthermore, mutation of the PVGF in iRhom2 to the *Drosophila* sequence PIGI abolished cleavage (Figure 2D). The converse experiment, mutating *Drosophila* iRhom from PIGI to the mammalian sequence PVGF, caused *Drosophila* iRhom to be cleaved (Figure 2D). Overall, these data confirm that the widely conserved (Figure S2C) PVGF motif in the luminal juxtamembrane region adjacent to TMD1 determines the primary cleavage of iRhom proteins.

As highlighted earlier, expression of N-terminally tagged full-length iRhom2 further revealed the generation of several smaller protein fragments (Figure 1D, indicated by triangle and hashtag symbols) which, based on their size, must lack TMD1. This indicates that the N-terminal fragment formed after primary SPC cleavage can undergo further processing. To investigate this secondary cleavage, we focused on the biggest of these fragments, sized around 40 kDa (Figure 1D, indicated by hashtag symbol). Internal deletions of several regions within iR2-1-403 indicated that amino acids 246–284 were required for the generation of this nuclear iRhom2 fragment (Figure 2E, white asterisks). Further analysis showed that this stretch of 38 amino acids contains three motifs highly conserved between human and mouse iRhom1 and iRhom2 (Figure S2D) and that deletion of amino acids 275-DVFESPPL-282 inhibited the formation of the major nuclear iRhom2 (Figure 2F, white asterisks), indicating that this sequence is necessary for the secondary cleavage event that generates soluble nuclear iRhom2. The presence of several smaller cleaved fragments was not affected by our deletions (Figures 2E and 2F), shown by empty triangle symbols, indicating that their generation was independent of that specific secondary cleavage site. *In silico* analysis using Procleave to identify predicted protease cleavage sites indicated that 275-DVFESPPL-282 contains several potential protease cleavage sites (Table S1). Treatment with (E64-d)—a broad-spectrum cysteine protease inhibitor, but not with Pepstatin A or AEBSF—inhibitors of other protease families partially inhibited the formation of the 40 kDa fragment (Figure S2E), suggesting a potential role of cysteine-type proteases. Additionally, expression of iRhom2 fragment (iR2-1-278) ending at the proposed secondary cleavage site 275-DVFE|SPPL-282 confirmed its exclusive localization in the nucleoplasm (Figure S2F).

Finally, we assessed whether iRhom2 mutants incapable of primary cleavage can fulfill two well-characterized iRhom2 functions: the destabilization of EGF-like ligands^8^ and the maturation of ADAM17.^7,9^ All uncleavable mutants were able to downregulate EGF protein levels indistinguishably from WT iRhom2, implying that these iRhom2 mutants are functional (Figure S2G). ADAM17 maturation was also unaffected by the uncleavable mutant PVGF → PIGI, implying that this process, too, was not dependent on iRhom2 cleavage (Figure S2H). ADAM17 maturation was, however, inhibited by the other uncleavable iRhom2 mutants (AI, L4, and L8) (Figure S2H). This apparently contradictory result is explained by co-immunoprecipitation assays, which show that those iRhom2 mutants defective in ADAM17 processing had markedly reduced binding between iRhom2 and ADAM17 (Figure S2I), whereas the PVGF → PIGI mutation binds normally. Overall, these results indicate that neither EGF degradation nor ADAM17 maturation depends on iRhom2 cleavage.

### SPC cleaves iRhom2

**Figure 3:**
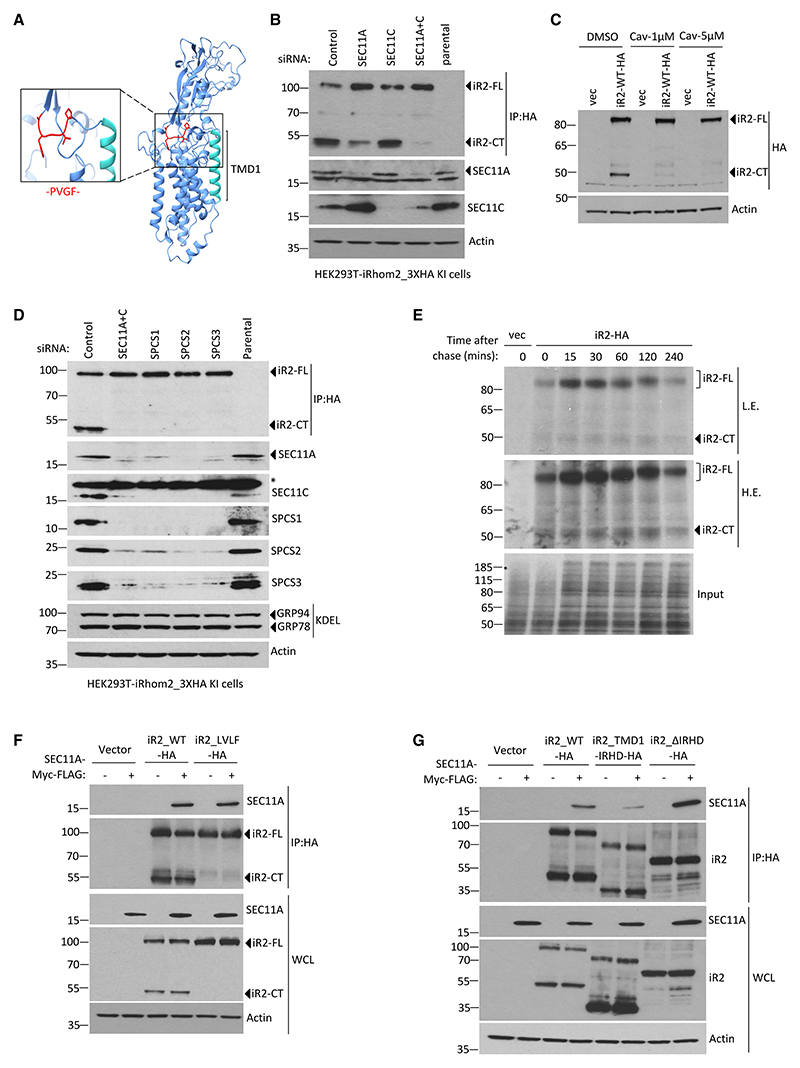
iRhom2 is cleaved by signal peptidase complex (A) Structure prediction for iRhom2 by AlphaFold. Region containing amino acids involved in iRhom2 cleavage (PVGF) is highlighted in zoomed box. (B) Endogenous iRhom2 proteins were detected by immunoblotting in HEK293T cells with knockin of 3XHA tag at C-terminal of iRhom2 gene locus, transfected with control siRNA and catalytic subunits SEC11A or SEC11B or both siRNAs. (C) iRhom2 proteins were analyzed in HEK293T cells transfected with iRhom2-3XHA in the presence of cavinafungin (Cav) at indicated doses by immunoblotting using HA antibody. (D) Endogenous iRhom2 proteins were detected in HEK293T cells with knockin of 3XHA tag at C-terminal of iRhom2 gene locus, transfected with control siRNA and components of SPC siRNAs. KDEL was used to detect GRP94 and GRP78 ER chaperone proteins. (E) C-terminally HA tagged iRhom2 proteins stably expressed in HEK293T cells were analyzed following ^35^S-Met radioactive pulse-chase labeling. Cells were pulsed for 4 min followed by chase at indicated time points. (F and G) Levels of wild-type iRhom2, mutant iRhom2, and SEC11A proteins were detected in HEK293T cells transfected with indicated constructs by immunoblotting in whole-cell lysate (WCL) and immunoprecipitated lysate (IP: HA).

We sought to identify the protease responsible for primary iRhom2 cleavage. Structural prediction by AlphaFold agreed with TMD predictions that the PVGF motif is located within the ER lumen, immediately adjacent to TMD1 (Figure 3A). Treatment with broad-spectrum protease inhibitors against serine, cysteine, and aspartic acid proteases did not significantly inhibit cleavage of iR-hom2 (Figure S3A). One possibility we checked was RHBDL4, a rhomboid protease, located in the ER and capable of cleaving within the luminal domains of its substrates,^25,26^ but its knock-down had no effect on iRhom2 cleavage (Figure S3B).

We have previously reported an iRhom2 interaction screen, in which one of the top hits was SEC11C,^13^ one of the two catalytic subunits of eukaryotic SPC.^21,27^ Although SPC has a well-established function in removing canonical signal peptides from proteins entering the ER, it also catalyzes the cleavage of multiple other signal peptide-like sequences.^28–30^ Knockdown of SEC11C alone did not have any significant effect on cleavage of endogenous iRhom2 in HEK293T cells, but silencing the alternative SPC catalytic subunit, SEC11A, had a slight effect (Figure 3B). Strikingly, the combined knockdown of SEC11A and SEC11C abolished the cleavage of endogenous (Figure 3B) or overexpressed iRhom2 (Figure S3C). Additionally, treatment of cells with cavinafungin, a specific inhibitor of SPC,^31^ blocked the cleavage of iRhom2 (Figure 3C) and reduced nuclear accumulation of iRhom2 as observed by cellular fractionation (Figure S3D). Cavinafungin treatment also inhibited the cleavage of iRhom1 (Figure S3E), confirming a conserved role of SPC in the cleavage of both mammalian iRhom proteins. In further support of the role of SPC, knockdown of any of the three essential accessory subunits (SPCS1, SPCS2, and SPCS3) also blocked iRhom2 cleavage (Figure 3D); note that knockdown of one sub-unit of SPC leads to depletion of the others, a phenomenon often seen in multi-subunit protein complexes.^32^ In contrast, none of these treatments altered the expression of ER chaperones GRP78 or GRP94, indicating that SPC knockdown was not causing more general defects in ER homeostasis. Consistent with iRhom2 being a substrate of SPC, two signal peptide prediction algorithms^33,34^ identified a potential SPC cleavage site in iRhom2 at the sequence PVGFA|QH, which matches the region we have experimentally determined to be vital for cleavage (Figures 2 and S3F). No cleavage sites were predicted around TMD1 of DERLIN1, another ER-localized rhomboid-like pseudo-protease protein (Figure S3F). In addition, pulse-chase analysis showed that radio-labeled cleaved fragment was already present after 4 min of pulse-labeling and that cleavage was not enhanced during the chase over 4 h (Figure 3E). These cleavage kinetics are consistent with the expected properties of SPC, which primarily cleaves substrates co-translationally.^22^ Finally, the SEC11A catalytic subunit coimmunoprecipitated with both WT iRhom2 and the uncleavable LVLF mutant (Figure 3F). Deletion of TMD2 → 7 of iRhom2 (iR2_TMD1_IRHD) but not the luminal IRHD (iR2_ΔIRHD) showed that this interaction was dependent on the TMD domains of iRhom2 (Figure 3G).

Having identified SPC as responsible for the primary cleavage of the iRhom N-terminal domains, we asked whether the intramembrane protease signal peptide peptidase (SPP) might be involved in the subsequent secondary cleavages described above. SPP cuts a number of released signal peptides after canonical SPC processing,^35^ although this appears not to be universal.^36^ Using the SPP inhibitor (Z-LL)_2_ ketone, we detected neither an effect on nuclear staining of iRhom2 (Figure S3G) nor any further cleavage of nuclear iRhom2 (Figure S3H), indicating that SPP is not responsible for the secondary cleavage that generates the soluble fragments of iR2-NT.

### Nuclear iRhom2 modifies the cellular transcriptome

**Figure 4:**
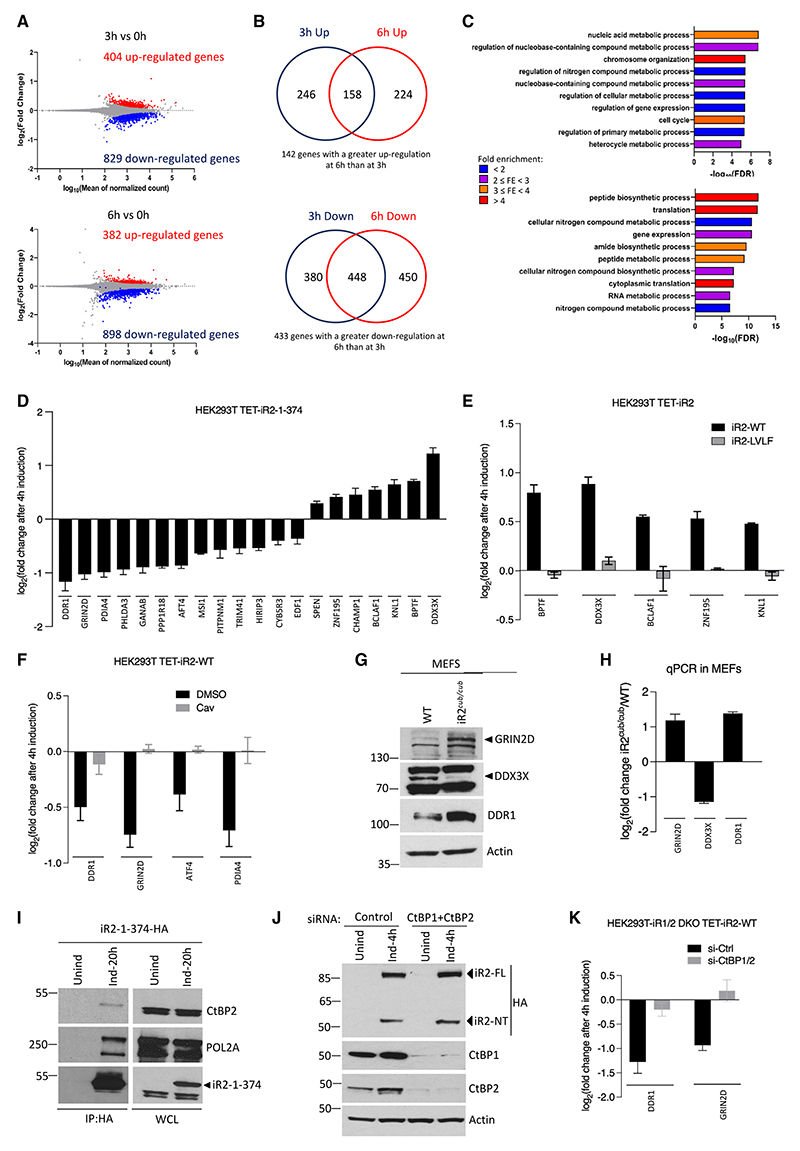
Nuclear iRhom2 induces gene expression changes (A) MA plot showing differentially expressed genes (upregulated in red, downregulated in blue) in HEK293T cells expressing inducible iRhom2-1-374 for 3 or 6 h against the 0 h control. n = 3, adjusted p < 0.05. (B) Venn diagrams showing overlap between significantly upregulated or downregulated genes at 3 and 6 h of iRhom2-1-374 induction. Genes with a greater downregulation (n = 433) or a greater upregulation (n = 142) at 6 than at 3 h of expression were considered more likely targets of nuclear iRhom2. (C) Bar graphs showing summary of the Gene Ontology (GO) enrichment analysis on the specific set of upregulated (n = 142) or downregulated (n = 433) genes, categorized as likely targets of nuclear iRhom2. FDR, false discovery rate. Different colors denote the range of fold enrichment (FE). (D) Graph showing validation of 20 potential target genes from RNA-seq data by quantitative RT-PCR in HEK293T cells expressing inducible iRhom2-1-374 for 4 h. Data are presented as log_2_-fold change relative to uninduced cells as mean ± SEM, n = 3. (E and F) Graphs showing transcript levels of selected target genes by qPCR in HEK293T cells expressing for 4 h either inducible wild-type (iR2-WT) or uncleavable iRhom2 (iR2-LVLF) (E) or inducible wild-type iRhom2 in the presence of cavinafungin (1 μm/18 h) (F). Data are presented as log_2_-fold change relative to uninduced cells as mean ± SEM, n = 3. (G and H) Protein levels (G) and transcript levels (H) of selected target genes of nuclear iRhom2 were analyzed in wild-type and iRhom2^cub/cub^ mouse embryonic fibroblasts (MEFs). qPCR data are presented as log_2_-fold change relative to wild-type cells as mean ± SEM, n = 3. (I) Levels of iRhom2, CtBP2, and TFIID proteins were detected in HEK293T cells expressing inducible iRhom2-1-374 by immunoblotting in whole-cell lysate (WCL) and immunoprecipitated lysate (IP: HA). (J and K) Levels of iRhom2 and CtBP proteins by immunoblotting (J) and transcript levels of indicated target genes by qPCR (K) were analyzed in HEK293T cells expressing inducible human HA-iRhom2 for 4 h and transfected with control siRNA and combined CtBP1/CtBP2 siRNAs. Data are presented as log_2_-fold change relative to uninduced cells as mean ± SEM, n = 3.

To ask whether the nuclear iRhom2 fragment has biological activity, we characterized the consequences of expressing the nuclear iR2-1-374 fragment (Figure 1E). We opted to use this fragment as it represents the largest domain of N-terminal iRhom2 that lacks TMD1 and is therefore exclusively located solubly in the nucleus. This experimental design comes with the caveat that some of the smaller secondary fragments highlighted in Figures 2E and 2F might not have the same functional characteristics. Nevertheless, our aim was to survey the full biological potential of soluble nuclear iRhom2. We generated HEK293T cells stably expressing HA-tagged iR2-1-374, and biochemical fractionation experiments detected iR2-1-374 in both soluble (S4) and insoluble, chromatin-containing (P4) nuclear fractions (Figure S4A). The soluble (S4) fraction was increased by MNase-mediated DNA digestion (Figure S4A), similar to control Histone H3 protein, indicating that nuclear iRhom2 associates with chromatin. We also generated HEK293T cells stably expressing HA-tagged iR2-1-374 under a tetracycline-inducible promoter (Figure S4B) and observed that iR2-1-374 co-immunoprecipitated with the B1 subunit of RNA polymerase II (RNA Pol II) and transcription factor IID (TFIID) (Figure S4C), both integral components of the eukaryotic gene transcription complex.^37^ To test the implication that nuclear iR2-1-374 might therefore influence gene expression, using RNA-seq, we examined the cellular transcriptome at 3 and 6 h after induction. The expression of 1,233 and 1,280 genes were significantly changed (adjusted p < 0.05) at 3 and 6 h, respectively (Figure 4A). At 3 h induction, 404 genes were upregulated, and 829 genes were downregulated. At 6 h induction, 382 genes were upregulated, and 898 genes were downregulated (Figure 4A). A complete list of differentially expressed genes is provided in Table S2.

We analyzed the genes that were either upregulated (158 genes) or downregulated (448 genes) at both 3 and 6 h time points and which showed a greater difference at 6 than at 3 h (Figure 4B). Using gene set enrichment analysis (GSEA),^38^ we identified nucleic acid metabolism, chromosome organization, regulation of gene expression, and cell cycle as the top processes associated with genes upregulated by iR2-1-374 (Figure 4C; Table S3) and peptide biosynthesis, translation, and RNA metabolism as the most prominent downregulated processes (Figure 4C; Table S3).

Quantitative real-time PCR validation of 20 differentially regulated genes confirmed that 7 were significantly upregulated by iR2-1-374, including *KNL1, BCLAF1, BPTF, DDX3X*, and *ZNF195* and 13 were significantly downregulated, including *GRIN2D, DDR1, ATF4, GANAB, PHLDA3*, and *PDIA4* (Figure 4D). The iR2-1-278 fragment, corresponding to the largest detectable iRhom2 soluble nuclear fragments (Figures 2E and 2F), similarly altered the expression of 11 of the 19 genes tested (Figure S4D). Importantly, blocking SPC cleavage of iRhom2 with the uncleavable LVLF mutant (Figure S4E) abolished the upregulation of several of these validated genes (Figure 4E). Further supporting the significance of SPC cleavage, treatment with cavinafungin inhibited SPC-mediated cleavage of iRhom2 in WT cells (Figure S4F) and prevented changes in gene expression (Figure 4F). Similar effects were observed using HEK293T-iRhom1/2 DKO cells expressing WT human iRhom2 under a tetracycline-inducible promoter^10^ (Figure S4G). Together, these data confirm the requirement for iRhom2 cleavage and the presence of nuclear iRhom2 for changes in target gene expression.

We addressed whether the transcriptional regulation of these genes by iR2-1-374 translated into changes at the protein level. We selected three genes that showed the most consistent regulation by WT iRhom2 and iR2-1-374 in HEK293T cells and examined their expression in WT and iRhom2^*cub/cub*^ mouse embryonic fibroblasts (MEFs). These mouse cells endogenously express a form of iRhom2 that contains a naturally occurring mutation that removes most of the cytoplasmic N-terminal domain.^20^ iRhom2^*cub/cub*^ cells showed the upregulation of GRIN2D and DDR1 proteins and downregulation of DDX3X protein (Figure 4G); they also showed corresponding changes in transcript levels (Figure 4H). These results indicate that the regulation of some target genes by nuclear iRhom2 is conserved between human and mouse cells.

As nuclear iRhom2 does not appear to be a TF, we investigated how it might regulate gene expression. Using the eukaryotic linear motif (ELM) prediction algorithm, we found in the N-terminal domain of iRhom2 a potential binding site for CtBP1 and CtBP2, which are nuclear factors that repress gene expression^39,40^ (Figure S4H). The PxDLS motif found in known nuclear-interacting proteins of CtBPs is conserved in iRhom2 (Figure S4I). Co-immunoprecipitation with iR2-1-374 pulled down CtBP2 and RNA Pol II (Figure 4I), supporting a possible functional interaction of CtBP2 with nuclear iRhom2. Knockdown of both CtBP1 and CtBP2 in HEK293T-iRhom1/2 DKO cells expressing inducible WT human iRhom2 (Figure 4J) markedly abrogated the downregulation of several genes by nuclear iRhom2 (Figure 4K). A similar effect was observed using MTOB, a chemical inhibitor blocking necessary dimerization of CtBP1 and CtBP2^41,42^(Figure S4J).

Together, these results support a biological role for the nuclear iRhom2 N-terminal domain in regulating gene expression and indicate that, at least in some cases, the downregulation of targets depends on CtBP1/2.

### Nuclear iRhom2 expression is enhanced in human skin pathologies

**Figure 5:**
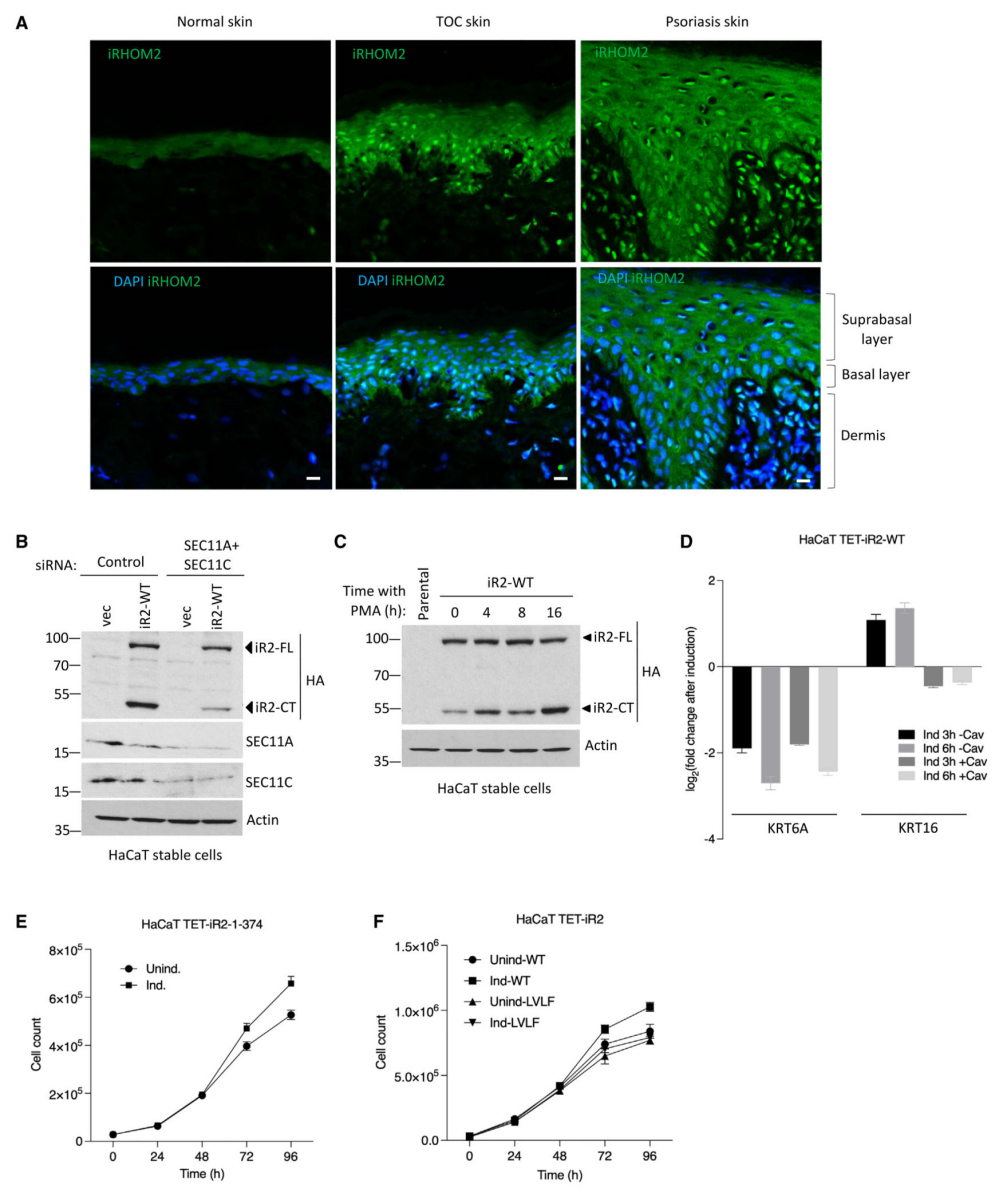
Human skin diseases show high levels of nuclear iRhom2 (A) Endogenous iRhom2 expression were determined by immunohistochemistry in the basal, suprabasal, and dermis layers in both TOC interfollicular and lesional psoriatic skin epidermis compared with control interfollicular skin from patient samples. Tissues were stained for iRhom2 (green) and DAPI (blue). n = 1 for each human tissue. Scale bars, 20 μm. (B and C) iRhom2, SEC11A, and SEC11B proteins were detected in HaCaT keratinocyte cells stably expressing iRhom2-3XHA after transfection with control siRNA or combined SEC11A and SEC11C siRNAs (B) or after treatment with 200 nM PMA for indicated time intervals (C). (D) Graphs showing transcript levels of keratin 6 (KRT6A) and keratin 16 (KRT16) by qPCR in HaCaT cells expressing inducible wild-type human iRhom2 (iR2-WT) for 4 h in the presence of cavinafungin (1 μm/18 h). Data are presented as log_2_-fold change relative to uninduced cells as mean ± SEM, n = 3. (E and F) Growth curves of HaCaT cells expressing inducible iRhom2-1-374 (E) wild-type iRhom2 (iR2-WT) or uncleavable mutant iRhom2 (iR2-LVLF) (F) over indicated time points. Data are presented as mean ± SEM, n = 3.

Beyond a well-characterized role in inflammatory signaling in macrophages, one of the main sites of iRhom2 expression is skin.^17^ Moreover, mutations in the iRhom2 N-terminal domain cause skin pathologies in the inherited disease tylosis with oesophageal cancer (TOC),^16,43^ and there have been other reported associations between iRhom2 and skin pathology.^44,45^ It was therefore striking to observe that patient normal skin samples showed a detectable expression of iRhom2 in the nuclei, which was elevated in the suprabasal and basal layers of TOC interfollicular skin by immunohistochemical staining (Figure 5A) and cellular fractionation (Figure S5A). This experiment was performed with an antibody against the N terminus of human iRhom2, validated using shRNA against iRhom2 in TOC cells (Figure S5B). Similar observations were made in the epidermis of lesional psoriatic skin (Figure 5A) and skin from the sole of the foot from diffuse non-epidermolytic palmoplantar keratoderma (NEPPK) patient biopsy (Figure S5C). Nuclear iRhom2 was also observed in immune cell populations residing within the dermis, particularly in lesional psoriatic skin biopsies (Figures 5A and S5C).

To explore the potential role of nuclear signaling of iRhom2 in skin, we stably expressed iRhom2 in HaCaT cells, a human epidermal keratinocyte line. As in HEK293T cells (Figure 3), knockdown of the catalytic subunits of SPC (SEC11A and SEC11C) inhibited the cleavage of iRhom2 (Figure 5B). Psoriasis is characterized by abnormal proliferation and differentiation of keratinocytes, combined with chronic inflammation,^46^ and treatment with the phorbol ester phorbol 12-myristate 13-acetate (PMA) is commonly used to model the epidermal thickening and dermal inflammation of psoriatic lesions.^47,48^ PMA treatment of iRhom2-expressing HaCaT and HEK293T cells with endogenous C-terminally tagged iRhom2 led to an increase in the cleavage of iRhom2 (Figures 5C and S5D). This enhanced cleavage was significantly abrogated by the knockdown of SPC catalytic subunits (Figure S5E) and cavinafungin (Figure S5F), indicating that PMA-induced cleavage of iRhom2 is mediated by SPC.

Addressing whether the TOC mutations had any direct effect on iRhom2 cleavage, we observed no difference in cleavage between WT and mutant human iRhom2 proteins containing various TOC mutations (UK-I186T and GER-P189L) (Figure S5G). We did find that, as previously reported,^20^ cycloheximide pulse-chase showed a modest but reproducible increase in the half-life of TOC mutant iRhom2 compared with WT (Figure S5H), although we have no direct evidence that this contributes to the elevated nuclear iRhom2 seen in TOC skin samples (Figure 5A).

Since keratin 16 (K16) and its type II binding partner keratin 6 (K6) have been reported to be differentially regulated in TOC keratinocytes compared with control cells,^44^ we assayed if these changes were dependent on nuclear iRhom2. Using HaCaT cells stably expressing WT full-length human iRhom2 under a tetracycline-inducible promoter, we confirmed a marked decrease in *KRT6A* (K6) and an increase in *KRT16* (K16) transcript levels (Figure 5D). Interestingly, treatment with cavinafungin had no effect on *KRT6A* but abolished the upregulation of *KRT16* by iRhom2 (Figure 5D). This indicates the role of cleaved iRhom2 in regulating *KRT16* expression and adds to evidence of a regulatory relationship between iRhom2 and K16.

Finally, to investigate a potential biological function of nuclear iRhom2, we used two cell systems, HaCaT cells expressing (1) nuclear iR2-1-374 or (2) full-length WT or the uncleavable mutant (LVLF) human iRhom2, under a tetracycline-inducible promoter. Expression of the nuclear iRhom2 N-terminal domain alone led to increased cell proliferation at 72 and 96 h, compared with control cells (Figure 5E). In addition, expression of full-length iRhom2 also led to increased cell proliferation, but this was not observed in cells expressing the uncleavable mutant (Figure 5F). Together, these results further indicate the biological significance of nuclear iRhom2 expression in the skin, and its potential regulation of processes that include cellular proliferation.

## Discussion

iRhoms, which are primarily located in the ER and the plasma membrane, are the best-studied non-protease members of the rhomboid-like superfamily.^1,5^ In this work, we have uncovered an iRhom-mediated signaling pathway, unrelated to their core function of membrane protein regulation via TMD recognition. Signaling is mediated by the proteolytic release of the iRhom N-terminal domain, which translocates to the nucleus, where it regulates gene expression. iRhoms join a select group of membrane proteins with a secondary nuclear function triggered by the proteolytic release of intracellular domains, of which the best-known examples include Notch, sterol regulatory element-binding protein (SREBP), and the ATF6 branch of the unfolded protein response.^49–51^

In each of the previously discovered examples, targets are cleaved by an intramembrane protease, which generates a fragment with too short a hydrophobic helix to be retained in the membrane. In the case of SPC cleavage of iRhom2, there must be a secondary cleavage to release the cleaved N-terminal domain from its membrane anchoring TMD. Indeed, we see that iRhom2 exists in two distinct nuclear sub-locations: membrane tethered in the nuclear envelope and chromatin bound or soluble in the nucleoplasm. We do not know the identity of the protease(s) responsible for the secondary cleavage, although we have mapped a secondary cleavage site and shown that this can be cut by cysteine-type proteases. This uncertainty reflects wider ignorance about the fate of canonical signal peptides cleaved by SPC. Some are degraded by the intramembrane aspartyl protease, SPP,^35^ but the fate of most signal peptides after SPC cleavage remains uncertain.^36^ In the case of iRhom2, we have experimentally ruled out SPP as the secondary protease that releases the soluble form of the N terminus.

SPC is primarily responsible for the removal of signal peptides from proteins entering the ER.^21,22^ Signal peptides are typically 15–30 amino acids long and reside in the first 30 amino acids of the coding sequence. Despite being associated with a TMD nearly 400 amino acids from the N terminus of the protein, the SPC cleavage site of iRhom2 broadly resembles the normal determinants of signal peptide cleavage^52,53^: two positively charged conserved amino acids (H and R) (the n-region), immediately preceding TMD1 (the h-region), followed by uncharged conserved amino acid segment (YGIAPVGF) (the c-region). At the predicted cleavage site of iRhom2 (PVGFA|Q), residues at positions −1 and −3 are Ala and Gly, respectively, and together with a Gln residue at the +1 position, these align with the consensus amino acids observed in eukaryotic signal peptidase cleavage sites.^54^ Other non-canonical cleavage of TMDs by SPC has been reported. For example, SPC removes 83 residues from the *Drosophila* cell surface protein Crumbs,^55^ 37 residues from the human cytomegalovirus protein UL40,^56^ and 135 residues from the canine distemper virus fusion glycoprotein F0.^57^ Most recently, Zanotti et al. have also reported that SPC has a widespread quality control function for many membrane proteins.^30^

It is essential that iRhom2 is only partially cleaved by SPC because other functions, like ADAM17 activation and response to ER stress, require the full-length protein. The mechanistic basis for this partial SPC cleavage of iRhom2, and how it is regulated, remains unknown, but it is notable that the timing and efficiency of signal sequence cleavage do vary for different SPC substrates. In the case of the HIV-1 gp160 envelope protein, for example, slow and inefficient signal sequence cleavage provides a checkpoint mechanism to ensure full folding, and maturation has occurred prior to onward trafficking from the ER.^58^ The flaviviridae family also takes advantage of slow processing by endogenous SPC, to ensure a correct sequence of cleavages of the viral polyprotein—necessary for efficient virus particle assembly and propagation.^59^ In both these cases, the cause of inefficient or slow processing has been mapped to non-canonical sequences in the SPC recognition sequence; hence, it will be interesting in the future to explore more widely the regulation and precise determinants of iRhom2 cleavage by SPC.

The regulation of the cellular transcriptome by nuclear iRhom2 is likely to be indirect as the N-terminal domain has no discernible features of a typical TF (e.g., transactivation or DNA-binding domains). Indeed, our observations that nuclear iRhom2 can bind to chromatin and interact with RNA Pol II and TFIID, components of the eukaryotic transcription complex,^37^ indicate that its regulation of gene expression is likely as part of a transcription activator or repressor complex. We have confirmed this in the specific case of iRhom2 binding to CtBP1/2, which we have shown to be necessary for downregulation of some of the iRhom2 targets. CtBPs act mainly by repressing gene expression through the recruitment of corepressor complexes.^39^ It would seem most likely that the soluble form of nuclear iRhom2-NT is responsible for its transcriptional regulatory function, but we note that it is also possible that the membrane-tethered nuclear iRhom2-NT could also play a direct role in gene expression changes. Nuclear envelope tethered proteins mostly act through interaction with chromatin-associated proteins,^60^ although LAP2β and MAN1 can bind directly to DNA.^61,62^ MAN1 can also act as a TF scavenger,^63,64^ sequestering R-Smads, regulators of transforming growth factor β (TGF-β), bone morphogenic protein (BMP), and activin signaling.^65^

Dominant iRhom2 mutations are the cause of the inherited syndrome TOC, which is characterized by palmoplantar keratoderma, oral precursor lesions, and a high lifetime risk of TOC.^16^ iRhom2^TOC^ mutants show upregulated shedding of EGF ligands by ADAM17,^19,43^ which is proposed to contribute to disease pathology. Our discovery of an unexpected nuclear function of the iRhom2 N-terminal domain suggests that iRhom2-dependent changes in gene expression may also contribute to pathogenesis. Consistent with this idea, we show a significant elevation of nuclear iRhom2 in three skin pathologies: TOC, psoriasis, and NEPPK. Importantly, unlike TOC, psoriasis and NEPPK are not associated with iRhom2 mutations, implying that elevated nuclear iRhom2 is not specific to the presence of TOC mutations. Although the potential pathogenic mechanisms need further exploration, it is striking that all three skin diseases exhibit epidermal keratinocyte hyperplasia^16,66,67^ and that epidermal thickness has been associated with iRhom2 expression in both mice and humans.^16,44,68,69^ In further support for the pathological relevance of nuclear iRhom2, we show that expression of nuclear iRhom2 (1) promotes cellular proliferation of keratinocytes cells and (2) upregulates the expression of Keratin 16, a cytoskeletal scaffolding protein associated with hyperproliferative states such as inflammation,^70^ wound healing,^71^ cancer,^72^ and physical stress.^44^ Nevertheless, we want to emphasize that there is much to learn about the potentially complex role of iRhom2 in skin pathologies. For example, as well as increased cell division, psoriasis is characterized by acute infiltration of inflammatory immune cells, including dendritic cells, macrophages, and T cells, which induce the release of chemokines and cytokines, including TNF.^67^ iRhom2 is highly expressed in immune cells and, through its canonical role in ADAM17 activation, controls TNF secretion.^7,9,73^ Overall, therefore, it seems likely that the pathogenesis of skin disease associated with iRhom2 may combine aspects of both inflammatory signaling and nuclear functions.

Finally, iRhoms represent an interesting example of the still rather poorly explored the phenomenon of pseudoenzymes regulating intracellular signaling pathways.^3,4^ Specifically, iRhoms are pseudoproteases, having lost through evolution the proteolytic activity of their more ancient cousins, the rhomboid intramembrane serine proteases. Until now, all known iRhom function has been apparently related to the proposed core function of members of the rhomboid-like superfamily, namely the specific recognition of TMDs and regulation of transmembrane proteins. Not only does this work expand our specific understanding of iRhom function and illustrate the modular nature of rhomboid-like proteins, but it also highlights how evolution can build completely new functions into pseudoenzymes, increasing functional divergence from their ancestral enzymes.

### Limitations of the study

Our study shows the generation of a nuclear form of iRhom2 following its cleavage by SPC, a protease best known for its role in removing signal sequences from proteins as they enter the ER. This raises mechanistic questions about how iRhom2 is recognized as a non-canonical substrate, and whether the proteolytic mechanism is the same as when cleaving signal peptides. As described above, further clarification is also needed about the identity of the secondary proteases that trim and release from the ER membrane the primary product of SPC cleavage. In assessing the pathological significance of nuclear iRhom2, the lack of gender and ethnic diversity in the patient biopsy samples means that further studies are needed to assess the generalizability of the prevalence of nuclear iRhom2 in these skin conditions. Furthermore, unlike TOC, psoriasis and NEPPK are not associated with iRhom2 mutations, implying that elevated nuclear iRhom2 is not specific to the presence of TOC mutations. We will therefore in the future want to understand the common factors between these skin diseases that lead to elevated nuclear iRhom2. Finally, we will also need to resolve the extent to which iRhom2 cleavage by SPC is a driver of disease in the skin (and potentially elsewhere) or a consequence of other pathological mechanisms.

## Star★Methods

### Key Resources Table

**Table:** 

REAGENT or RESOURCE	SOURCE	IDENTIFIER
Antibodies		
Mouse anti-β-actin	Santa Cruz Biotechnology	Cat#sc-47778; RRID:AB_626632
Mouse anti-FLAG(R) M2-Peroxidase (HRP)	Sigma-Aldrich	Cat#A8592; RRID:AB_439702
Rat anti-HA-Peroxidase High Affinity (clone 3F10)	Roche	Cat#12013819001; RRID:AB_390917
Mouse anti-KDEL	AbCam	Cat#ab12223; RRID:AB_298945
Rabbit anti-iRhom2-NT-specific	Adrain et al.^7^	RRID:AB_3076550
Rabbit anti-RHBDF2	Sigma-Aldrich	Cat#SAB1304414; RRID:AB_3076552
Goat anti-Myc	AbCam	Cat#ab9132; RRID:AB_307033
Rabbit anti-SEC11A	Proteintech	Cat#14753-1-AP; RRID:AB_2186230
Rabbit anti-SEC11C	Novus Biologicals	Cat#NBP1-80774; RRID:AB_11009401
Rabbit anti-SPCS1	Proteintech	Cat#11847-1-AP; RRID:AB_2195400
Rabbit anti-SPCS2	Sigma-Aldrich	Cat#HPA013386; RRID:AB_1857426
Mouse anti-SPCS3	Santa Cruz Biotechnology	Cat#sc-377334; RRID:AB_3076547
Rabbit anti-RHBDL4	Fleig et al.^25^	RRID:AB_3076551
Mouse anti-RNA Polymerase II CTD	MBL International	Cat#MABI0601; RRID:AB_2728735
Rabbit anti-TFIID	Santa Cruz Biotechnology	Cat#sc-273; RRID:AB_2200059
Rabbit anti-Histone H3 (D1H2) XP	Cell Signaling Technology	Cat#4499; RRID:AB_10544537
Rabbit anti-NMDAR2D (GRIN2D)	Novus Biologicals	Cat#NBP2-94573; RRID:AB_3076548
Rabbit anti-CtBP1 (D2D6)	Cell Signaling Technology	Cat#8684; RRID:AB_10859907
Rabbit anti-CtBP2	Cell Signaling Technology	Cat#13256; RRID:AB_2798164
Rabbit anti-DDR1 (D1G6)XP	Cell Signaling Technology	Cat#5583; RRID:AB_10694842
Rabbit anti-DDX3 (D19B4)	Cell Signaling Technology	Cat#8192; RRID:AB_10860416
Rabbit anti-ADAM17	AbCam	Cat#ab39162; RRID:AB_722565
Mouse anti-GAPDH	AbCam	Cat#ab8245; RRID:AB_2107448
Rabbit anti-Integrin Alpha V	Proteintech	Cat#27096-1-AP; RRID:AB_2880753
Rabbit anti-Lamin A	AbCam	Cat#ab26300; RRID:AB_775965
Rabbit anti-HA Tag (C29F4)	Cell Signaling Technology	Cat#3724; RRID:AB_1549585
Mouse anti-HA.11	Enzo Life Sciences	Cat#ENZ-ABS120-0200; RRID:AB_3076549
Mouse anti-BAP31	Enzo Life Sciences	Cat#ALX-804-601-C100; RRID:AB_2050797
Chicken anti-GFP	AbCam	Cat#ab13970, RRID:AB_300798
Rabbit anti-SUN2	Sigma-Aldrich	Cat#HPA001209; RRID:AB_1080465
Goat Anti-rabbit IgG, HRP-linked	Cell Signaling Technology	Cat#7074; RRID:AB_2099233
Horse Anti-mouse IgG, HRP-linked	Cell Signaling Technology	Cat#7076; RRID:AB_330924
Mouse Anti-goat IgG, HRP-linked	Santa Cruz Biotechnology	Cat#sc-2354; RRID:AB_628490
Goat Anti-chicken IgG, HRP-linked	Novus Biologicals	Cat#NB7303; RRID:AB_524727
Bacterial and virus strains		
Stellar™ Competent Cells	Takara	Cat#636766
Chemicals, peptides, and recombinant proteins		
DAPI	Thermo Fisher Scientific	Cat#D1306
Cycloheximide	Sigma-Aldrich	Cat#C7698
Doxycycline	MP Biomedicals	Cat#SKU0219504405
DMEM medium	Sigma-Aldrich	Cat#D6429
L-Glutamine (200 mM)	Thermo Fisher Scientific	Cat#25030024
10% FBS	Thermo Fisher Scientific	Cat#10500064
MG-132	Sigma-Aldrich	Cat#474791
Chloroquine diphosphate salt	Sigma-Aldrich	Cat#C6628
3-MA	Sigma-Aldrich	Cat# 189490
E-64d	Sigma-Aldrich	Cat# E8640
Pepstatin A	Sigma-Aldrich	Cat# P5318
AEBSF, Hydrochloride	Sigma-Aldrich	Cat#101500
3,4-Dichloroisocoumarin	Sigma-Aldrich	Cat#D7910
Phorbol-12-myristate-13-acetate	Sigma-Aldrich	Cat#P8139
Cavinafungin	Estoppey et al.^31^	N/A
(Z-LL)_2_ Ketone	Sigma-Aldrich	Cat#SML1442
MTOB sodium	MedChemExpress	Cat#HY-135046
Opti-MEM™ I Reduced-Serum Medium	Thermo Fisher Scientific	Cat#31985070
Blasticidin S	Sigma Aldrich	Cat#15205
Zeocin	Thermo Fisher Scientific	Cat#R25001
Puromycin	Thermo Fisher Scientific	Cat#A1113803
cOmplete™, EDTA-free Protease Inhibitor Cocktail	Roche	Cat#4693132001
Micrococcal Nuclease (MNase)	Sigma Aldrich	Cat#N3755
HBSS	Thermo Fisher Scientific	Cat#D14025092
EasyTag™ EXPRESS^35^S Protein Labeling Mix [^35^S]-	PerkinElmer	Cat#NEG772007MC
DMEM, high glucose, no methionine, no cysteine	Thermo Fisher Scientific	Cat#D21013024
PNGase F	New England Biolabs	Cat#P0704S
Endo H	New England Biolabs	Cat#P0702S
VECTASHIELD anti-fade mounting medium	Vectorlabs	Cat#H-1000-10
Immu-Mount	Thermo Fisher Scientific	Cat#9990402
Critical commercial assays		
Q5 High-Fidelity DNA Polymerase	New England Biolabs	Cat#M0491S
InFusion HD cloning Kit	Takara Bio	Cat#639649
FuGENE HD Transfection reagent	Promega	Cat#E2312
Lipofectamine™ RNAiMax Transfection reagent	Thermo Fisher Scientific	Cat#13778075
Pierce™ Coomassie (Bradford) Protein Assay Kit	Thermo Fisher Scientific	Cat#23236
Amersham ECL Western Blotting Detection Reagent	Cytiva	Cat#RPN2106
SuperSignal™ West Pico PLUS Chemiluminescent Substrate	Thermo Fisher Scientific	Cat#34577
Pierce™ Anti-HA Magnetic beads	Thermo Fisher Scientific	Cat#88837
Concanavalin A Sepharose beads	Sigma Aldrich	Cat#C9017
Cell Fractionation Kit	Cell Signaling Technology	Cat#C9038
Moxi Z™Mini Automated Cell Counter	Orf low	Cat#MXZ001
Direct-zol™ RNA MiniPrep Plus kit	Zymo Research	Cat#R2072
SuperScript™ VILO™ cDNA synthesis Kit	Thermo Fisher Scientific	Cat#11754050
UltraScript™ cDNA Synthesis Kit	PCRBiosystems	Cat#PB30.11-10
TaqMan™ Gene Expression Master Mix	Applied Biosystems	Cat#4369016
Deposited data		
RNA-seq raw data	This study	GEO accession code: GSE219008
Immunoblots and Immunofluorescence raw data	This study	Mendeley Dataset: https://doi.org/10.17632/ssdn55787d.1
Experimental models: Cell lines		
HEK293T cells	ATCC	Cat#CRL-3216; RRID:CVCL_0063
HaCaT cells	ATCC	Cat#PCS-200-011; RRID:CVCL_0038
Wild-type MEF cells	Christova et al.^17^	N/A
iRhom1/2 DKO MEF cells	Christova et al.^17^	N/A
Wild-type MEF cells (cub pair)	Siggs et al.^74^	N/A
iRhom2^cub/cub^ MEF cells	Siggs et al.^74^	N/A
Wild-type keratinocyte cells	Blaydon et al.^16^	N/A
iRhom2-TOC keratinocyte cells	Blaydon et al.^16^	N/A
HEK293T-iRhom1/2 DKO cells	Kunzel et al.^13^	N/A
HEK293T-vector stable cells	Kunzel etal.^13^	N/A
HEK293T-iRhom2-3XHA stable cells	Kunzel et al.^13^	N/A
HEK293T-iRhom2-3XHA CRISPR knockin cells	Kunzel et al.^13^	N/A
HEK293T-iR2-1 -374-3XHA stable cells	This study	N/A
HEK293T-iR2-WT-3XHA TET-inducible cells	This study	N/A
HEK293T-iR2-LVLF-3XHA TET-inducible cells	This study	N/A
HEK293T-iR2-1 -374-3XHA TET inducible cells	This study	N/A
HEK293T-iR1/2 DKO + 3XHA-iR2-WT TET-inducible cells	Dulloo et al.^10^	N/A
HaCaT-iR2-3XHA stable cells	This study	N/A
HaCaT-3XHA-iR2-WT TET-inducible cells	This study	N/A
HaCaT-3XHA-iR2-LVLF TET-inducible cells	This study	N/A
HaCaT-3XHA-iR2-1-374 TET-inducible cells	This study	N/A
iR1/2 DKO-iR2-WT-3XHA stable MEF cells	This study	N/A
iR1/2 DKO-iR2-L4-3XHA stable MEF cells	This study	N/A
iR1/2 DKO-iR2-L8-3XHA stable MEF cells	This study	N/A
iR1/2 DKO-iR2-LVLF-3XHA stable MEF cells	This study	N/A
iR1/2 DKO-iR2-PIGI-3XHA stable MEF cells	This study	N/A
iR1/2 DKO-iR2-A1-3XHA stable MEF cells	This study	N/A
Experimental models: Organisms/strains		
Wild-type mice (C57BL/6J)	Adrain et al.^7^	RRID:IMSR_JAX:000664
iRhom2 KO mice (C57BL/6J)	Adrain et al.^7^	RRID:IMSR_JAX:000664
Oligonucleotides		
TaqMan™ gene expression assay probes	Thermo Fisher Scientific	Details in Table S4
ON-TARGETplus SMARTpool human siRNA for SEC11A	Dharmacon	Cat#L-006038-00-0005
ON-TARGETplus SMARTpool human siRNA for SEC11C	Dharmacon	Cat#L-046035-01-0005
ON-TARGETplus SMARTpool human siRNA for SPCS1	Dharmacon	Cat#L-020577-00-0005
ON-TARGETplus SMARTpool human siRNA for SPCS2	Dharmacon	Cat#L-020897-00-0005
ON-TARGETplus SMARTpool human siRNA for SPCS3	Dharmacon	Cat#L-010124-00-0005
ON-TARGETplus SMARTpool human siRNA for CTBP1	Dharmacon	Cat#L-008609-00-0005
ON-TARGETplus SMARTpool human siRNA for CTBP2	Dharmacon	Cat#L-008962-00-0005
Non-targeting siRNA control	Dharmacon	Cat#D-001206-13-50
Stealth siRNA against RHBDF2	Thermo Fisher Scientific	Cat#HSS128594
Stealth siRNA against RHBDF2	Thermo Fisher Scientific	Cat#HSS128595
Stealth siRNA against RHBDL4	Thermo Fisher Scientific	Cat#HSS125697
Stealth siRNA against RHBDL4	Thermo Fisher Scientific	Cat#HSS125698
Stealth siRNA Negative Control	Thermo Fisher Scientific	Cat#12935300
shRNA against RHBDF2	Maruthappu et al.^44^	N/A
Recombinant DNA		
pCMV6-SEC11A-Myc-FLAG	Origene	Cat#RC204971
pCDNA3.1-3XMyc-EGF	Zettl et al.^8^	N/A
pEGFP-N1-iR1-HA-WT	Zettl et al.^8^	N/A
pEGFP-N1-iR2-HA-WT	Zettl et al.^8^	N/A
pEGFP-N1-iR2_DIRHD-HA	Dulloo et al.^10^	N/A
pEGFP-N1-iR2_TMD1_IRHD-HA	Dulloo et al.^10^	N/A
pM6P.Blast-iR2-WT-HA	Adrain et al.^7^	N/A
pLVX-TetONE-Puro/Zeo	Michael van der Weijer (Dunn School, Oxford)	N/A
pCMV-VSV-G (lentiviral infection)	Adrain et al.^7^	N/A
pCMV-dR8.91(lentiviral infection)	Adrain et al.^7^	N/A
pCL.10A1(retroviral infection)	Adrain et al.^7^	N/A
All other plasmids generated	This study	Details in Table S4
Software and algorithms		
ImageJ	NIH	https://imagej.nih.gov/ij/
Clustal Omega	EMBL-EBI	https://www.ebi.ac.uk/Tools/msa/clustalo/
SignalP 4.1	Petersen et al.^34^	https://services.healthtech.dtu.dk/services/SignalP-4.1/
PrediSi	Hiller et al.^33^	http://www.predisi.de/
Prism 9.4.0	GraphPad	https://www.graphpad.com/scientific-software/prism/
Cutadapt v1.18	Martin.^75^	N/A
STAR v2.7.3	Dobin et al.^76^	N/A
DESeq2 V1.30.1	Love et al.^77^	N/A
Apelgm V1.18.0	Zhu et al.^78^	N/A
Gene Ontology	Ashburner et al.^79^	N/A

### Resource Availability

#### Lead contact

Further information and requests for resources and reagents should be directed to and will be fulfilled by the lead contact, Matthew Freeman (matthew.freeman@path.ox.ac.uk).

#### Materials availability

All unique reagents generated in this study are available from the lead contact without restriction.

### Experimental Model And Study Participant Details

#### Human samples

Skin biopsies were obtained from patients undergoing surgery with written informed consent and approved by the Barts Health NHS Trust ethics committee (IRAS Project ID: 08/H1102/73). The study protocol conforms to the ethical guidelines of the Declaration of Helsinki. Human samples were biopsies from either of the two rare autosomal dominant skin conditions (NEPPK and TOC), and the other from a psoriatic lesion. The NEPPK skin biopsy was from a White female of unknown age with the following heterozygous change in *Aquaporin 5* gene: WT/p.Ile45ser. The TOC skin biopsy was from a White female of unknown age with the following heterozygous change in the *RHBDF2* gene: WT/p.Ile186Thr. The psoriasis lesional biopsy came from an anonymised individual of White ethnicity (gender and age unknown).

#### Mouse studies

Organs from wild-type and iRhom2 knockout mice (C57BL/6J) previously generated,^7,17^ were collected from sacrificed 16-week-old female animals and stored on dry ice or at −80°C. Tissues were lysed in Triton X-100 RIPA buffer (1% Triton X-100, 150 mM NaCl, 50 mM Tris-HCl (pH 7.5), 0.1% SDS, 0.5% sodium deoxycholate) supplemented with cOmplete™, EDTA-free Protease Inhibitor Cocktail (Roche, #4693132001) using a tissue homogeniser (Omni International). Lysates were cleared from cell debris by centrifugation (20,000 g, 4°C, 10 min) and used for immunoblotting. All procedures on mice were conducted in accordance with the UK Scientific Procedures Act (1986) under a project license authorized by the UK Home Office Animal Procedures Committee, project licenses 80/2584 and 30/2306, and approved by the Sir William Dunn School of Pathology Local Ethical Review Committee.

#### Cell culture

Mouse embryonic fibroblasts (MEFs) were isolated from Rhbdf1^−/−^/Rhbdf2^−/−^ (referred to as iRhom1/2 DKO) E13.5 embryos and wild-type C57BL/6J controls and immortalised by lentiviral transduction with SV40 large T antigen as previously described.^7,17^ Mouse embryonic fibroblasts (MEFs) cells from iRhom2^cub/cub^ and wild-type littermates have previously been described.^74^ Wild-type and TOC keratinocyte cell lines have previously been described.^16^ Human embryonic kidney (HEK293T) (#CRL-3216) and human epidermal keratinocyte (HaCaT) (#PCS-200-011) cells were previously obtained from ATCC, and HEK293T-iRhom1/2 DKO parental cells and cells stably expressing inducible human HA-iRhom2 has previously been described.^10,13^ Details for cell lines generated in this study are provided in the key resources table. All cells were cultured in high-glucose DMEM (Sigma-Aldrich, #D6429) supplemented with 10% fetal bovine serum (FBS) (Thermo Fisher Scientific, #10500064) and 5 mM glutamine (Gibco, #11539876) at 37°C with 5% CO_2_.

### Method Details

#### Molecular cloning and plasmids

Wild-type iRhom1, iRhom2 and mutants iRhom2_ ΔIRHD and iRhom2_TMD1-IRHD plasmids in pEGFP-N1 vector with a C-terminal HA tag have been previously described.^7,8,10^ N-terminal HA tagged constructs for iRhom1 and iRhom2 generated in this study were amplified from their corresponding cDNA by PCR and cloned with into either pEGFP-N1 (without EGFP protein) or pCDNA3.1 mammalian expression vectors. All human iRhom2 constructs derive from isoform 1 of the *RHBDF2* gene. Wild-type iRhom2 and *Drosophila* iRhom were cloned by PCR into pEGFP-N1 vector with a C-terminal FLAG tag. GFP-iRhom2-HA construct was generated by cloning of iRhom2-HA into pEGFP-N1 mammalian expression vector, in frame with EGFP protein at the N-terminal. Wild-type and mutant iRhom2 cDNAs were also cloned by PCR into pM6P.Blasticidin (for retroviral infection) and into pLVX-TetOne Puro/Zeo vectors (Clontech, #631849) (for lentiviral infection). SEC11A-Myc-FLAG was obtained from OriGene Technologies (#RC204971). Details of all plasmids used are provided in Table S4.

All cloning PCR was done using Q5 High-Fidelity DNA Polymerase (New England Biolabs, #M0491S) and InFusion HD cloning kit according to manufacturer’s protocol (Takara Bio, #639649). Site-directed mutagenesis (SDM) was done by PCR method and DpnI digestion. All constructs were verified by Sanger sequencing (Source Bioscience, Oxford, UK).

#### Drug treatments

The following drugs were used: Cycloheximide (Sigma Aldrich, #C4859), Doxycycline (Sigma Aldrich, #D9891), MG-132 (Sigma Aldrich, #474791), Chloroquine (Sigma Aldrich, #C6628), 3-MA (Sigma Aldrich, #189490), E-64d (Sigma Aldrich, #E8640), Pepstatin A (Sigma Aldrich, #P5318), AEBSF (Sigma Aldrich, #101500), 3,4-Dichloroisocoumarin (3,4 DCI) (Sigma Aldrich, #D7910), phorbol 12-myristate 13-acetate (PMA) (Sigma Aldrich, #P8139), Cavinafungin (gift from Martin Spiess), Z-LL_2_ Ketone (Merck Life Science, #SML1442), MTOB (MedChemExpress, #HY-135046). All drug concentrations are indicated in figure legends and in respective methods sections. Unless indicated, all drugs were incubated for 18-24h.

For cycloheximide chase assay, HEK293T cells transfected with indicated plasmids after 24h were treated with 100 μg/ml and harvested at indicated time points.

#### Transfection and transduction of cell lines

HEK293T cells transiently transfected with DNA in Opti-MEM™ | Reduced-Serum Medium (Thermo Fisher Scientific, #31985070) using FuGENE HD Transfection reagent (Promega, #E2312) and protein expression was analysed 24–48 h post transfection.

For knockdown experiments, siRNA was transfected using Lipofectamine RNAiMax Transfection reagent (Thermo Fisher Scientific, #13778075) according to the manufacturer’s protocol. ON-TARGETplus SMARTpool human siRNA (Dharmacon) for SEC11A (#L-006038-00-0005), SEC11C (#L-046035-01-0005), SPCS1(#L-020577-00-0005), SPCS2(#L-020897-00-0005), SPCS3 (#L-010124-00-0005), CTBP1 (#L-008609-00-0005), CTBP2 (#L-008962-00-0005), and RHBDF2 (#HSS128594, #HSS128595, Thermo Fisher Scientific), RHBDL4 (#HSS125697, #HSS125698, Thermo Fisher Scientific), and non-targeting siRNA control (Dharmacon: #D-001206-13-50, Thermo Fisher Scientific: Stealth RNAi™ siRNA Negative Control: #12935300) were used. Protein expression was analysed 72 h post siRNA transfection. For shRNA knockdown of iRhom2 in TOC keratinocytes, sequences and procedure used were previously described.^44^

MEFs, HEK293T and HaCaT cells stably expressing wild-type or mutant iRhom2 proteins were generated by retroviral transduction using pM6P.Blast retroviral constructs as previously described.^10,13^ In brief, HEK293T cells were transfected with indicated gene expressed in pM6P.Blast constructs together with packaging plasmid pCL.10A1. Viral supernatants for individual constructs were harvested after 48 h, cleared by centrifugation at 20,000 × g for 20 min, and co-incubated with HEK293T or HaCaT cells in the presence of 5 μg/ml polybrene and cells were selected with 10 μg/ml Blasticidin S (Sigma Aldrich, #15205). HEK293T, HEK293T-iRhom1/2 DKO, and HaCaT cells expressing either wild-type or mutant iRhom2 proteins under TET-inducible cells were generated by lentiviral infection using gene cloned into pLVX-TetOne Puro/Zeo vectors (gift from Michael van der Weijer-Dunn School, Oxford). Methodology is similar to retroviral transduction, with exception of packaging vectors (pCMV-VSV-G and pCMV-dR8.91) and selected with 2 μg/ml puromycin (Thermo Fisher Scientific, #A1113803) or 100 μg/ml Zeocin™ (Thermo Fisher Scientific, #R25001). Details of cell lines generated are provided in Table S4.

#### Antibodies

For immunoblotting and co-immunoprecipitation: Actin (Santa Cruz, #sc-47778; 1:5000), FLAG-HRP (Sigma Aldrich, #A8592; 1:4000), HA-HRP (Roche, #12013819001; 1:2000), KDEL (AbCam, #ab12223; 1:2000), mouse iRhom2-NT-specific (^7^; 1:500), iRhom2 (Sigma-Aldrich, #SAB1304414; 1:500), Myc (Abcam, #ab9132; 1:2000), SEC11A (Proteintech, #14753-1-AP; 1:500), SEC11C (Novus Biologicals, NBP1-80774; 1:500), SPCS1 (Proteintech, #11847-1-AP; 1:500), SPCS2 (Merck Life Science, #HPA013386; 1:500), SPCS3 (Santa Cruz, sc-377334; 1:500), RHBDL4 (^25^; 1:1000), POL2 (MBL International, MABI0601; 1:1000), TFIID (Santa Cruz, #sc-273; 1:1000), Histone H3 (Cell Signaling, #4499T; 1:4000), GRIN2D (Novus Biologicals, NBP2-94573; 1:1000), CTBP1 (Cell Signaling, # 8684S; 1:500), CTBP2 (Cell Signaling, # 13256S; 1:1000), DDR1 (Cell Signaling, #5583T; 1:1000), DDX3 (Cell Signaling, # 8192S; 1:1000), ADAM17 (AbCam, #ab39162; 1:2000), GAPDH (AbCam, #ab8245; 1:1000), ITGAV (Proteintech, #27096-1-AP, 1:1000), LAMIN A (AbCam, #ab26300; 1:4000).

For immunofluorescence/immunohistochemistry: DAPI (Thermo Fisher, #D1306; 1 μg/ml), HA (Cell Signaling Technology, #3724; 1:500), HA.11 (Enzo Life Sciences, #ABS120-0200; 1:500), BAP31 (Enzo Life Sciences, #ALX-804-601-C100; 1:250), GFP (AbCam, #ab13970; 1:500), iRHOM2 (Sigma-Aldrich, #SAB1304414; 1:50), SUN2 (Atlas Antibodies, HPA001209; 1:250)

#### SDS-PAGE and immunoblotting

Cells were washed with ice-cold PBS and then lysed on ice in Triton X-100 lysis buffer (1 % Triton X-100, 200 mM NaCl, 50 mM TrisHCl pH 7.4) supplemented with cOmplete™, EDTA-free Protease Inhibitor Cocktail (Roche, #4693132001). For detection of endogenous iRhom2 in keratinocytes, cells were lysed using lysis buffer (1 M Tris, 2.5 M NaCl, 10% glycerol, 0.5 M glycerophosphate, 1% Tween-20, 0.5% Nonidet P-40 and cOmplete™, EDTA-free Protease Inhibitor Cocktail) on ice followed by the freeze-thaw method. Cell lysates were cleared by centrifugation at 21,000 x g for 20 min at 4°C. Protein concentrations were measured using Pierce™ Coomassie (Bradford) Protein Assay Kit (Thermo Fisher Scientific, #23236). Cell lysates were denatured at 65°C for 15 min and ran either on 4-12 % NuPAGE™ Bis-Tris gels or 8-16 % Tris-Glycine Novex™ WedgeWell™ (Thermo Fisher Scientific, #NP0321, #XP0816A) in MOPS or Tris-Glycine running buffer respectively. PageRuler™ Plus Prestained Protein Ladder (Thermo Fisher Scientific, #26620) was used for protein molecular weight marker. Note this ladder runs differently on Bis-Tris and TriGlycine gels, resulting in different molecular weights according to manufacturer. Both types of gel were used throughout study and gels were transferred onto polyvinylidene difluoride (PVDF) membranes (Millipore, #IPVH85R). The membrane was blocked in 5 % milk-TBST (150 mM NaCl, 10 mM Tris-HCl pH 7.5, 0.05 % Tween 20, 5 % dry milk powder) before incubation with indicated primary and species-specific HRP-coupled secondary antibodies. All primary antibodies were made in 5% BSA-TBST except for HRP-conjugated antibodies. Band visualisation was achieved with Amersham ECL Western Blotting Detection Reagent (Cytiva, #RPN2106) or SuperSignal™ West Pico PLUS Chemiluminescent Substrate (Thermo Fisher Scientific, #34577) using X-ray film. Quantification of blots was done using Fiji (Image J).

#### Co-immunoprecipitation

HEK293T cells were transfected in 6-cm plates with indicated plasmids for 36-48 h before harvest. MEFs cells stably expressing iRhom2 were also processed according to the following steps. Cells were lysed on ice in Triton X-100 lysis buffer (1 % Triton X-100, 200 mM NaCl, 50 mM Tris-HCl pH 7.4) processed similarly as described in immunoblotting section above. Lysates were immunoprecipitated with 15μl pre-washed Pierce™ Anti-HA Magnetic beads (Thermo Fisher Scientific, #88837) at 4°C overnight on a rotor. Beads were washed 4-5 times with Triton X-100 wash buffer (1% Triton X-100, 500 mM NaCl, 50 mM Tris-HCl pH 7.4) and proteins were eluted by incubation at 65°C for 15 min in 23 SDS sample buffer.

For concanavalin A pull-down, N-glycosylated proteins were enriched by incubating cell lysates containing protease inhibitor and 1,10-phenanthroline (Sigma-Aldrich, #131377) with 20μl concanavalin A Sepharose beads (Sigma-Aldrich, # C9017) at 4°C for at least 2 h with rotation. Beads were washed with Triton X-100 wash buffer and proteins were eluted in 2x NuPAGE™ LDS sample buffer (Thermo Fisher Scientific, #NP008) supplemented with 50 mM DTT and 50 % sucrose for 15 min at 65°C and were ran on 4-12 % NuPAGE™ Bis-Tris gels.

#### Subcellular biochemical fractionation assays

For isolation of soluble nuclear and chromatin containing fractions (Figure S4A), process was performed as previously described.^80^ 2 × 10^7^ cells were harvested and resuspended in 500 μl buffer A (10 mM HEPES [pH 7.9], 10 mM KCl, 1.5 mM MgCl_2_, 0.34 M sucrose, 10% glycerol, 1 mM dithiothreitol, and protease inhibitor cocktail). Triton X-100 was added (0.1% final concentration), the cells were incubated on ice for 8 min, and nuclei (fraction P1) were collected by centrifugation (5 min, 1,300 × g, 4°C). The supernatant (fraction S1) was clarified by high-speed centrifugation (5 min, 20,000 × g, 4°C), and the super-natant (fraction S2) was collected. The P1 nuclei were washed once in bufferA and lysed for 30 min in buffer B (3 mM EDTA, 0.2 mM EGTA, 1 mM dithiothreitol, and protease inhibitor cocktail), and insoluble chromatin (fraction P3) and soluble (fraction S3) fractions were separated by centrifugation (5 min, 1,700 × g, 4°C). The P3 fraction was washed once with buffer B and was resuspended in a solution containing 10 mM Tris, 10 mM KCl, and 1 mM CaCl_2_, with or without 1 U of MNase (Sigma Aldrich, #3755). After 15 min incubation at 37°C, the reaction was stopped with EGTA (1 mM final concentration). Soluble and insoluble components were then separated by centrifugation (5 min, 1,700 × g, 4°C). The pellet was resuspended in sample buffer and sonicated before immunoblotting.

For isolation of total, membrane and nuclear fractions (Figure S3D), process was performed using Cell Fractionation Kit (Cell Signaling, #9038) as per manufacturer’s protocol.

For isolation of cytoplasm/membrane and nuclear fractions from control and TOC keratinocytes (Figure S5A), process was performed from 80% confluent 10-cm tissue culture dishes. Cells were rinsed with PBS at room temperature, scraped into ice-cold low salt buffer [10 mM Tris-HCl pH 7.5, 1 mM MgCl_2_, 10 mM KCl, plus protease inhibitor cocktail, incubated on ice for 15 min, and sheared with a 27-gauge needle until all the cells are lysed and left on ice for 20 min. After pelleting by centrifugation (800 *g*, 5 min, 4°C), supernatant A was removed into a fresh tube and keep on ice and pelleted nuclei were reconstituted in a high-salt lysis buffer (40 mM HEPES pH 7.5, 450 mM NaCl, 1 mM EDTA, plus PIC) and twice incubated on ice for 10 min followed by 1 min vortex prior to ice water bath sonication (8 cycles total; 1 cycle, 30 s on, 30 s off) and centrifugation (17,000 *g*, 10 min, 4°C) to pellet insoluble nuclear debris. The resulting supernatant represented the nuclear fraction. Supernatant A was then subjected to centrifugation (10,000 *g*, 10 min, 4°C) and transfer the supernatant to a fresh tube and keep on ice and this was representing the cytoplasm and membrane fraction and used for immunoblotting.

#### Radioactive pulse-chase labeling

HEK293T cell stably expressing C-terminally tagged iRhom2-3XHA were seeded in 60mm tissue culture dishes to a 90% confluency. Assay was done according to this protocol.^81^ In brief, cells were washed with 2 ml wash buffer (HBSS) (Thermo Fisher Scientific, #D14025092), followed by incubation with 2 ml of depletion medium [cysteine and methionine-free tissue-culture medium (Thermo Fisher Scientific, #D21013024) containing 10 mM HEPES, pH 7.4) for 15 min at 37°C in a humidified 5% CO_2_ incubator. Depletion medium was aspirated before adding 400 μL labelling medium [cysteine and methionine-free tissue-culture medium containing

10 mM HEPES, pH 7.4 + 100 μCi EasyTag™ EXPRESS^35^S Protein Labeling Mix [^35^S]-(PerkinElmer, Cat#NEG772007MC) per sample at 37°C] to the dish and incubated for 4 min pulse period. 2 ml of chase medium (complete tissue-culture medium containing 10 mM HEPES, 5 mM cysteine, 5 mM methionine, 37°C) was added at the end of pulse interval to stop labelling and incubated at desired chase intervals in a 37°C humidified 5% CO_2_ incubator. Chase medium was aspirated before addition of 2 mL ice cold stop buffer (HBSS, 4°C), and placed on ice. Cells were washed again with ice cold stop buffer just before addition of 600 μL ice-cold triton X-100 lysis buffer and incubated on ice for 20 min. Cells were scrapped and transferred to Eppendorf tubes and centrifuged at 21,000g for 20 min at 4°C. Lysates were immunoprecipitated as described in above section with anti-HA magnetic beads. Eluates were ran on 4-12 % NuPAGE™ Bis-Tris gels, which were dried on filter paper and exposed to film at -80°C.

#### Deglycosylation assay

Cells were lysed in Trition X-100 lysis buffer as described above. Lysates were first denatured with Glycoprotein Denaturing Buffer at 65°C for 15 min and then treated with Endoglycosidase H (Endo H) (New England Biolabs, #P0702S) or Peptide-*N*-Glycosidase F (PNGase F) (New England Biolabs, #P0704S) following the manufacturer’s instructions.

#### Cell proliferation assay

Cells were seeded at an original density of 2×10^4^ in 12-well plates in triplicates for each condition. Cells density was remeasured 24 h later to find the exact starting cell numbers (T=0 h), and subsequently harvest every 24h up to T=96 h. Cell were counted using Moxi Z Mini Automated Cell Counter (Orflow, #MXZ001) and data plotted using Graphpad Prism.

#### Quantitative real-time PCR

RNA was isolated from cells using Direct-zol™ RNA MiniPrep Plus kit (Zymo Research, #R2072) according to the manufacturer’s instructions and reverse transcribed using the SuperScript™ VILO™ cDNA synthesis Kit (Thermo Fisher Scientific, #11754050) or UltraScript™ cDNA Synthesis Kit (PCRBiosystems, #PB30.11-10). Resulting cDNA was used for quantitative real-time PCR (qPCR) using the TaqMan™ Gene Expression Master Mix (Applied Biosystems, #4369016). A list of all TaqMan gene expression assay probes used is provided in Table S4. For quantification, the relative quantity of samples was calculated according to the comparative Δ Ct method and normalized to GAPDH or ACTIN. Gene expression was compared to the corresponding wild-type or uninduced control.

#### Immunofluorescence and confocal microscopy

HEK293T cells were plated on 13-mm glass coverslips in 12-well plates and transfected with 100-250 ng of indicated constructs for 48 h prior to fixation. Cells were washed 2 times with PBS and fixed with 4% paraformaldehyde in PBS at room temperature for 20 min. Cells were then washed 3 times with PBS and permeabilised in 0.3% TX-100 in PBS for 20 min. Cells were blocked with 3% BSA in 50 mM Tris pH 7.5 for 30 min after removal of permeabilisation buffer. Cells were incubated overnight with indicated antibodies in blocking buffer at 4°C and then washed 3 times in permeabilisation buffer for (5 min each wash). Coverslips were incubated with corresponding species-specific fluorescently coupled secondary antibodies (Invitrogen) for 30 min. Cells were subsequently washed 4 times with PBS (5 μg/ml DAPI was added in second to last wash), prior to mounting on glass slides with VECTASHIELD® anti-fade mounting medium (Vectorlabs, #H-1000-10). Images were acquired with a laser scanning confocal microscope (Fluoview FV1000; Olympus) with a 6031.4 NA oil objective and processed using Fiji (Image J).

#### Immunohistochemistry

Immunohistochemistry was performed on frozen tissue; sections were air-dried before processed. Tissues were fixed in 4% paraformaldehyde (PFA) at room temperature for 15 min. If PFA fixation was used, samples were permeabilized with 0.1% Triton X-100. Tissues were washed three times with PBS for 5 min each and incubated with 5% goat serum in PBS for 1 h at room temperature to reduce nonspecific binding. Tissues were incubated with primary antibody for iRHOM2 (Sigma-Aldrich, #SAB1304414; 1:50) in 5% goat serum overnight at 4°C. The following day tissues were washed three times with PBS and incubated with the secondary antibody conjugated with Alexa Fluor (Molecular Probes) in 5% goat serum for 1 h at room temperature. After three washes, sections were incubated for 10 min with DAPI (100 ng/ml). Tissues were mounted onto slides using Immu-Mount (Thermo Fisher Scientific, #9990402). Fluorescence was evaluated in one single plane by Zeiss 710 confocal microscopy (Carl Zeiss).

#### RNA-seq

HEK293T cells stably expressing inducible iRhom2-1-374 were treated with 200 ng/ml of doxycycline for 0h, 3h and 6h. Triplicate samples were harvested, and RNA was extracted using Direct-zol™ RNA MiniPrep Plus kit (Zymo Research, #R2072) according to the manufacturer’s instructions. PolyA library preparation and RNA sequencing was performed by Novogene (UK) Company Ltd. Paired-end 150 bp sequencing was performed using Illumina NovaSeq 6000 sequencing system.

#### RNA-seq data processing and analysis

RNA-seq data were analysed as previously described.^82^ Adapters were trimmed with Cutadapt version 1.18^75^ in paired-end mode with the following options: –minimum-length 10 -q 15,10 -j 16 -A GATCGTCGGACTGTAGAACTCTGAAC -a AGATCGGAAGAGCACACGTC TGAACTCCAGTCAC. The remaining rRNA reads were removed by mapping the trimmed reads to the rRNA genes defined in the human ribosomal DNA complete repeating unit (GenBank: U13369.1) with STAR version 2.7.3^76^ and the parameters –runThreadN 16 –readFilesCommand gunzip -c -k –outReadsUnmapped Fastx –limitBAMsortRAM 20000000000 –outSAMtype BAM SortedByCoordinate. The unmapped reads were mapped to the human GRCh38.p13 reference sequence with STAR version 2.7.3a and the ENCODE parameters: –runThreadN 16 –limitBAMsortRAM 20000000000 –outSAMtype BAM SortedByCoordinate –quantMode GeneCounts –outFilter-MultimapNmax 20 –outFilterType BySJout –alignSJoverhangMin 8 –alignSJDBoverhangMin 1 –outFilterMismatchNmax 999 –alignIn-tronMin 20 –alignIntronMax 1000000 –alignMatesGapMax 1000000.

The number of aligned reads per gene obtained with STAR –quantMode GeneCounts were used to perform the differential expression analysis with DESeq2 version 1.30.1^77^ and apeglm version 1.18.0.^78^ For the iRhom2-1-374 RNA-seq dataset, an adjusted p-value < 0.05 was considered statistically significant. We considered as more likely iRhom2-1-374 target genes those found differentially expressed at both 3 h and 6 h and with either a greater downregulation at 6 h than 3 h or a greater upregulation at 6 h than at 3 h.

Gene ontology (GO) enrichment analysis was performed with the Gene Ontology resource website (^79^; Gene Ontology, 2021). MA plots, heatmaps, and GO plots were produced with GraphPad Prism 9.4.0.

### Quantification and Statistical Analysis

All statistical analyses were performed with GraphPad Prism 9.4.0. p-value for RNA-Seq data were analysed by the Wald test, as described in the DESeq2 package.^77^ Unless indicated, all immunofluorescence and immunoblotting data are representative of 2-3 independent experiments. Value of n and details are provided in figure legends. The data are expressed as the mean ± standard error of mean (SEM).

## Data Availability

RNA-sequencing data generated during this study are available in the Gene Expression Omnibus repository, https://www.ncbi.nlm.nih.gov/geo (accession GEO: GSE219008) and will be publicly available upon publication. Original imaging and immunoblotting data are available at Mendeley Data (https://doi.org/10.17632/ssdn55787d.1). This paper does not report original code. Any additional information required to re-analyse the data reported in this paper is available from the lead contact upon request.

## Highlights

- iRhom2 pseudoprotease is cleaved by signal peptidase complex (SPC)
- Cleaved N-terminal cytoplasmic domain of iRhom2 translocates to the nucleus
- Nuclear iRhom2 affects gene expression
- Increased level of nuclear iRhom2 is associated with different skin pathologies

## In brief

iRhoms are membrane proteins that regulate signaling by recognizing client transmembrane domains. Dulloo et al. reveal an additional mechanism of action: upon cleavage by signal peptidase, an intracellular fragment of iRhom2 translocates to the nucleus, modulating gene expression. Nuclear iRhom2 promotes the growth of keratinocytes and accumulates in skin pathologies.

## Footnotes

**Supplemental information**
Supplemental information can be found online at https://doi.org/10.1016/j.molcel.2023.12.012.
